# Pyo-Pneumo-Thorax

**Published:** 1877-11

**Authors:** E. V. Stoddard


ART. II.—*Pyo-Pneumo-Thorax. Treatment by aspiration, and permanent drainage Tube.* By Prof. E. V. STODDARD, M. D.

Wm. A. Smith, aet. 22, was admitted to Rochester City Hospital, March 13, 1876; by occupation a file cutter. Father died of "brain difficulty," and Mother of cancer of stomach.

He has enjoyed fair health until two years since, when he commenced coughing. His best weight has been 155 lbs., present weight 145 lbs. Has never suffered from Hæmoptysis. One year since had pneumonia. One week ago was taken with chill, since which time he has been confined to his bed. Had been at work at his occupation till this attack. At present his condition is anæmic, pulse 136, respiration short, quick and frequent. Has night sweats, bowels loose; and considerable pain in right side and through epigastrium. Tongue clean and pale.

Physical examination of chest reveales a very interesting condition. *Percussion* over right side gives marked dullness over upper fourth of that lung, amounting to flatness in the lower three-fourths. By auscultation, tubular respiration appears over apex, with no respiratory sounds over lower three-fourth of the lung. Over left lung, in upper third, slight modification of percussion note occurs in a lessened resonance; vesicular murmur exaggerated and expiration prolonged; heart pushed downwards and over towards the left axilla. Succussion of the patient produces a splashing sound, *so distinct as to be heard by any one standing near the bed.*

Diagnosis—Hydro-Pneumo-Thorax.

As his condition was one of considerable exhaustion, from car travel and walking, he was fed liberally, and stimulants with Quinine (gr. iij. every four hours,) were ordered. To quiet his cough and relieve pain,

℞. Aceti Opii m x.

Spts Chloroformi m xx—P. R. N.

March 20th.—Having rallied considerably, it was decided to remove the fluid from the chest. This was accordingly done with the aspirator. The needle was inserted in the 8th intercostal space, on a line drawn perpendicularly from the center of the axilla. By the means ℥.c x v j (or 7¼ pints) of purulent fluid were removed from the pleural cavity. This gave the patient great relief, and was followed by an abatement of all his urgent symptoms. His improvement in every respect was remarkable.

March 31st.—Evidences of a re-accumulation of the fluid in the chest becoming more and more marked, it was decided to open the chest wall by puncture, insert a silver tube and thus keep the pleural cavity constantly free from purulent fluid. Accordingly an incision was made, in the site of the former puncture, in the 8th intercostal space, and a silver tube introduced, which could be closed with a small cork. By this operation ℥ lxxxvij of pus (5.$\frac{7}{16}$ pints) was removed. The tube was kept in place by an elastic band around the body. No pains were taken to prevent the ingress of air, as the communication of the chest cavity with the interior of the lungs was evident, and the presence of air in the pleural cavity could be demonstrated, by causing the patient to cough, by which effort air was forced out through the tube.

April 3d.—Removed the silver tube and inserted a rubber tube $\frac{1}{8}$ inch calibre and about three feet in length, terminating in a bottle containing a weak solution of carbolic acid. About three and a half ounces of pus were discharged daily. *The chest cavity was washed out each day with warm water, containing a little carbolic acid, by using the tube and bottle as a syphon:* Elevating the bottle above the patient filling the chest, and placing the bottle on the floor withdrawing the fluid.

April 6th.—Pleuritic friction sound appeared over upper part of left lung. Dry cups were applied, and quinine freely given. He slowly improved from this time until August 5th, when his general condition became much worse. Diarrhœa was troublesome, and cough more frequent and severe. He continued in this condition, with gradually increasing indications of disorganization of the left lung, through the fall and winter. The flow of pus from the side, through the tube continued, and, gradually failing, he died March 6th, 1877, one year and three days after coming under observation.

The *post mortem* examination was held eighteen hours after death.

On opening the chest, the right pleural cavity, over its whole surface, resembled the walls of a large abscess; no healthy tissue being found in its lining membrane. The lung occupied about one-tenth of the cavity of the pleura; it was compressed along the spinal column. It was quite hard and firm, with an occasional indication of tubercle.

The tube was found to have been inserted into the lowest part of the pleural cavity.

The *left lung* was tuberculous throughout, and was undergoing rapid degeneration; very little of its tissue was available for respiration.

The *Heart* was healthy but slightly enlarged.

The *Liver* enlarged and fatty.

In closing this imperfect history, I would call attention to

1st. The large amount of purulent fluid removed at one time by aspiration, ($7\frac{1}{4}$ pints or $12\frac{11}{16}$ pints by two operations within eleven days).

2d. The long time which the rubber tube was worn, ($11\frac{1}{2}$ months).

3d. The *washing out of the chest each day*, and the ready means for doing so offered by the rubber tube and bottle.

4th. The prolongation of life and great relief to the patient by this mode of treatment.

